# Supplementary material for: The Systems Biology Research Tool: evolvable open-source software
Source: BMC Syst Biol. 2008 Jun 29;2:55. doi: 10.1186/1752-0509-2-55 (PMC2446383; doi:10.1186/1752-0509-2-55)
Supplement: Additional file 1 — SBRT Archive. An archive of the current version of the Systems Biology Research Tool. [file 1752-0509-2-55-S1.zip › sbrt-1.4.0/doc/developers_guide/api/sbrt/kernel/math/EquivTester.html]

EquivTester


|  |  |  |
| --- | --- | --- |
| **Overview** \| **Package** \| **Class** \| **Use** \| **Tree** \| **Deprecated** \| **Index** \| **Help** | |  |
| **PREV CLASS**   **NEXT CLASS** | **FRAMES**    **NO FRAMES**     **All Classes** |  |
| SUMMARY: NESTED \| FIELD \| CONSTR \| METHOD | DETAIL: FIELD \| CONSTR \| METHOD |  |


---


## sbrt.kernel.math Interface EquivTester<T>

**Type Parameters:**: `T` - the class type.

**All Known Implementing Classes:**: DoubleEquiv, IntervalEquiv

---

``` public interface EquivTester<T> ```

This interface is used to represent *equivalence testers*.

**Author:**
:   This interface was written by Jeremiah Wright while in the
    Wagner lab.

---

**Method Summary**

`<A extends T,B extends T>
  
boolean`

`areEquiv(A o1,
B o2)`
  
          Tests the provided objects for equivalence.

**Method Detail**

### areEquiv

```
<A extends T,B extends T> boolean areEquiv(A o1,
                                           B o2)
```

:   Tests the provided objects for equivalence.

    :   **Type Parameters:**: `A` - a type extending T.: `B` - a type extending T. **Parameters:**: `o1` - an object.: `o2` - another object. **Returns:**: `true` if the provided objects are equivalent; `false` otherwise.


---


|  |  |  |
| --- | --- | --- |
| **Overview** \| **Package** \| **Class** \| **Use** \| **Tree** \| **Deprecated** \| **Index** \| **Help** | |  |
| **PREV CLASS**   **NEXT CLASS** | **FRAMES**    **NO FRAMES**     **All Classes** |  |
| SUMMARY: NESTED \| FIELD \| CONSTR \| METHOD | DETAIL: FIELD \| CONSTR \| METHOD |  |


---
